# Supplementary material for: Modular actin nano-architecture enables podosome protrusion and mechanosensing
Source: Nat Commun. 2019 Nov 15;10:5171. doi: 10.1038/s41467-019-13123-3 (PMC6858452; doi:10.1038/s41467-019-13123-3)
Supplement: Supplementary file 2 — Description of Additional Supplementary Files [file 41467_2019_13123_MOESM2_ESM.pdf]

**Description of Additional Supplementary Files**

File Name: Supplementary Movie 1
Description: Assembling podosome in a DC transfected for cortactin-BFP, α-actinin-tagRFP, vinculin-GFP and LifeactiRFP. Image series were acquired on a Leica DMI6000 with 1 image per 6 s. Playback speed: 15 fps

File Name: Supplementary Movie 2
Description: DCs were transfected with Lifeact-GFP and vinculin-mCherry and seeded on stiff substrates. Image series were acquired on a Zeiss LSM880 with Airyscan settings with 1 image per 15 s. Playback speed: 15 fps

File Name: Supplementary Movie 3
Description: DCs were transfected with Lifeact-GFP and vinculin-mCherry and seeded on soft substrates. Image series were acquired on a Zeiss LSM880 with Airyscan settings with 1 image per 15 s. Playback speed: 15 fps

File Name: Supplementary Movie 4
Description: twSTICS reveals vinculin and actin flows in podosome clusters in a DC seeded on stiff PDMS.

File Name: Supplementary Movie 5
Description: twSTICS reveals vinculin and actin flows in podosome clusters in a DC seeded on soft PDMS.

File Name: Supplementary Movie 6
Description: DCs were transfected with Lifeact-GFP and seeded on rhodamine-gelatin labeled stiff substrates. Image series were acquired on a Leica DMI6000 with 1 image per 5 min. Playback speed: 15 fps
