# Supplementary material for: Modular actin nano-architecture enables podosome protrusion and mechanosensing
Source: Nat Commun. 2019 Nov 15;10:5171. doi: 10.1038/s41467-019-13123-3 (PMC6858452; doi:10.1038/s41467-019-13123-3)
Supplement: Supplementary file 9 — Supplementary Information [file 41467_2019_13123_MOESM9_ESM.pdf]

Supplementary Figures

Modular actin nano-architecture enables podosome protrusion and mechanosensing

Van den Dries et al.

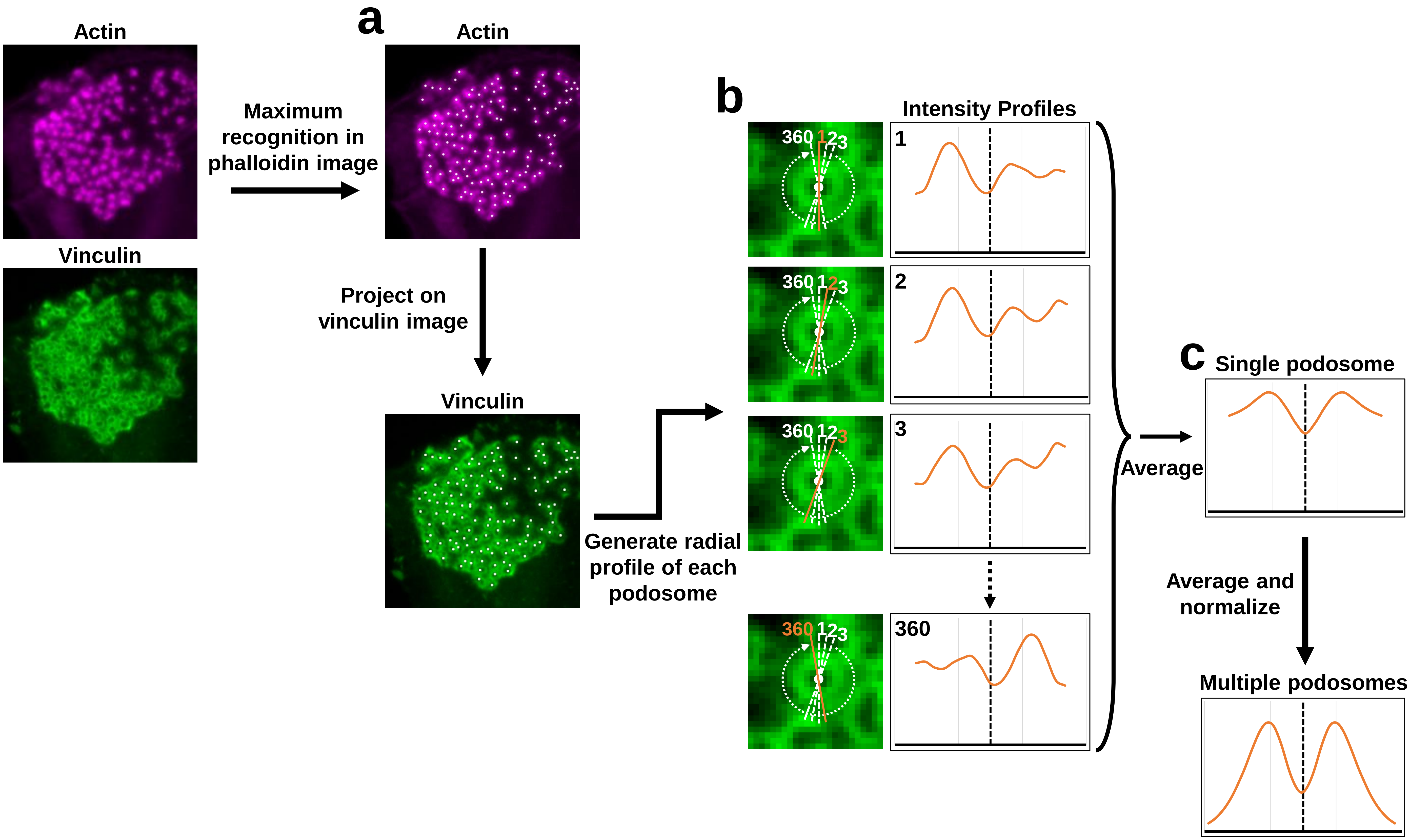

Supplementary Figure 1

Overview of the radial fluorescence profile analysis

To quantify the localization of each of these proteins with respect to the podosome core we used a semi-automatic self-developed ImageJ macro that **a**) recognizes the podosome core centers based on the actin image **b**) draws a vertical line of ~3um through the center of the core that rotates around its center and collects a profile for every line and **c**) produces an average radial intensity profile as a function of distance from the podosome core center. Profiles are normalized to the minimum and maximum for visualization and comparison. Of note, for some of the panels we only present one half of the intensity profile since the radial intensity profiles are symmetric by definition.

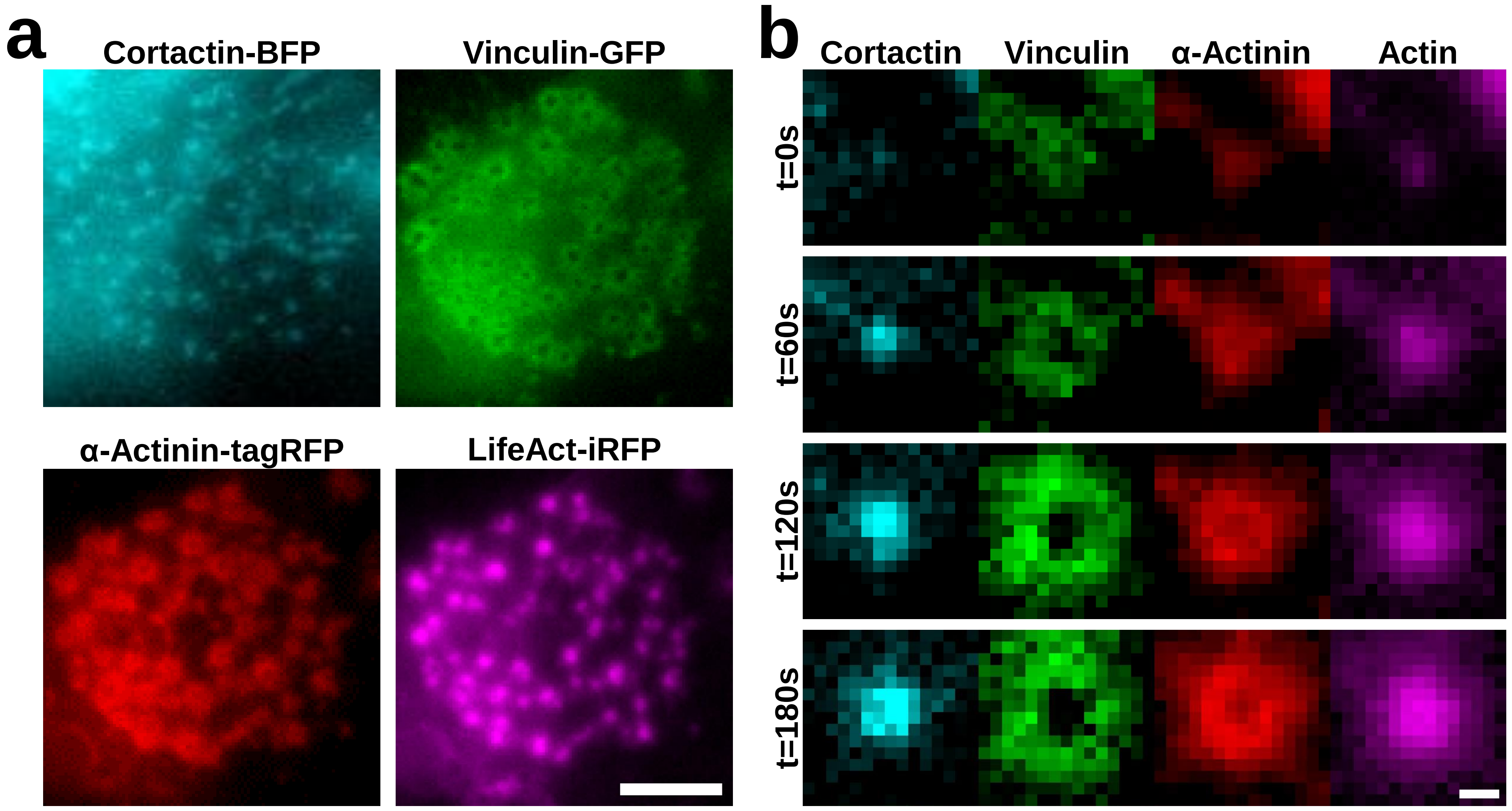

Supplementary Figure 2

Simultaneous visualization of different actin-binding proteins at podosomes in living cells
a-b, Widefield images of a DC transfected with cortactin-BFP (cyan), vinculin-GFP (green), α-actinin-tagRFP (red) and LifeAct-iRFP (magenta). **a,** shows a still from an entire podosome cluster and **b,** shows a few stills in time of a single assembling podosome. Scale bars: **a** = 5 µm, **b** = 0.5 µm

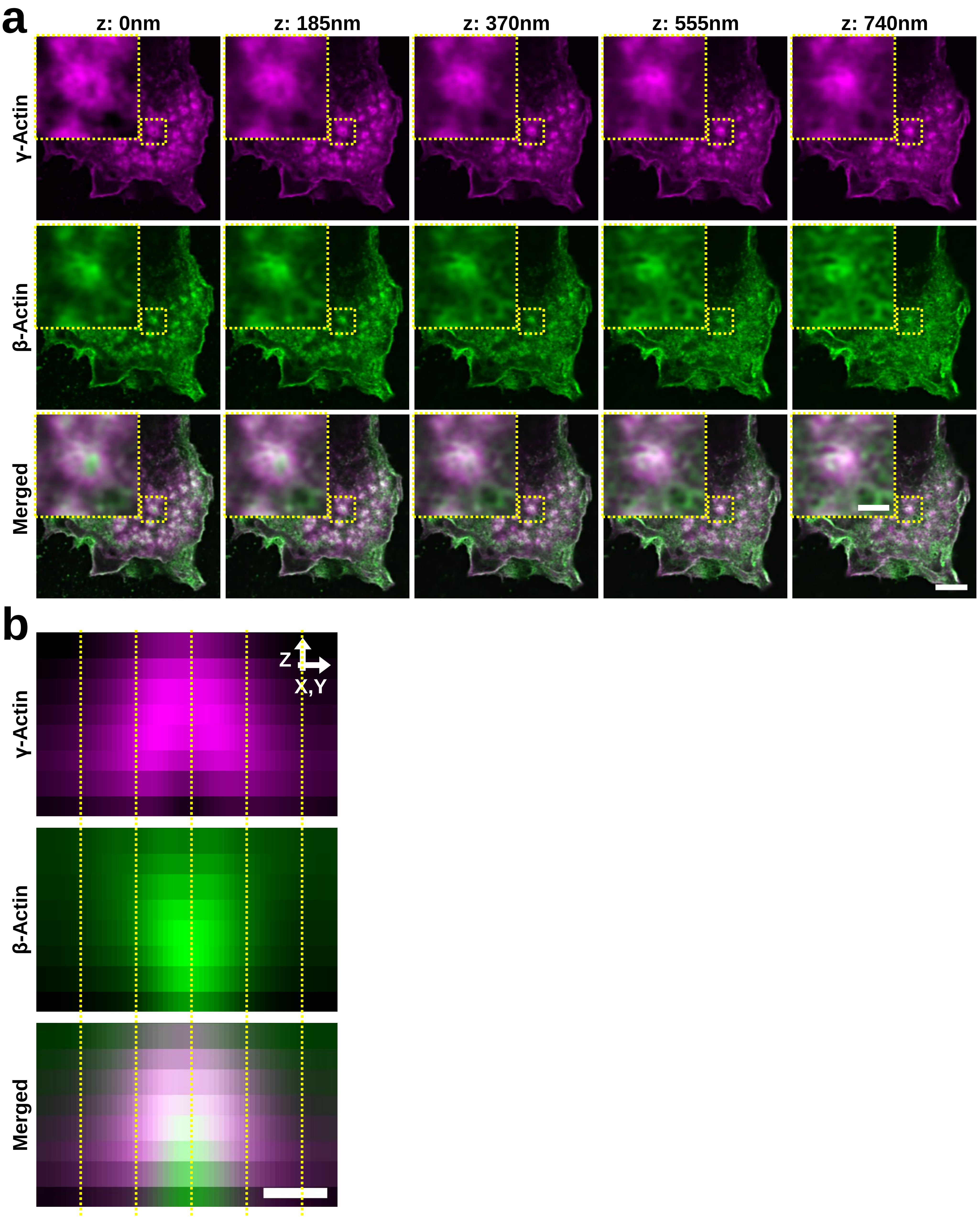

Supplementary Figure 3

γ and β-actin isoforms differentially localize to cPM and pPM within podosomes of murine BMDCs
a, 3D-Airyscan images of a murine BMDC stained for γ (magenta) and β-actin (green). Insets depict a single podosome. **b,** Average radial orthogonal view of γ (magenta) and β-actin (green) in 10 podosomes. Bottom panel shows the merged image. Scale bars: **a** = 2 µm, **b** = 0.5 µm; insets: 0.5 µm.

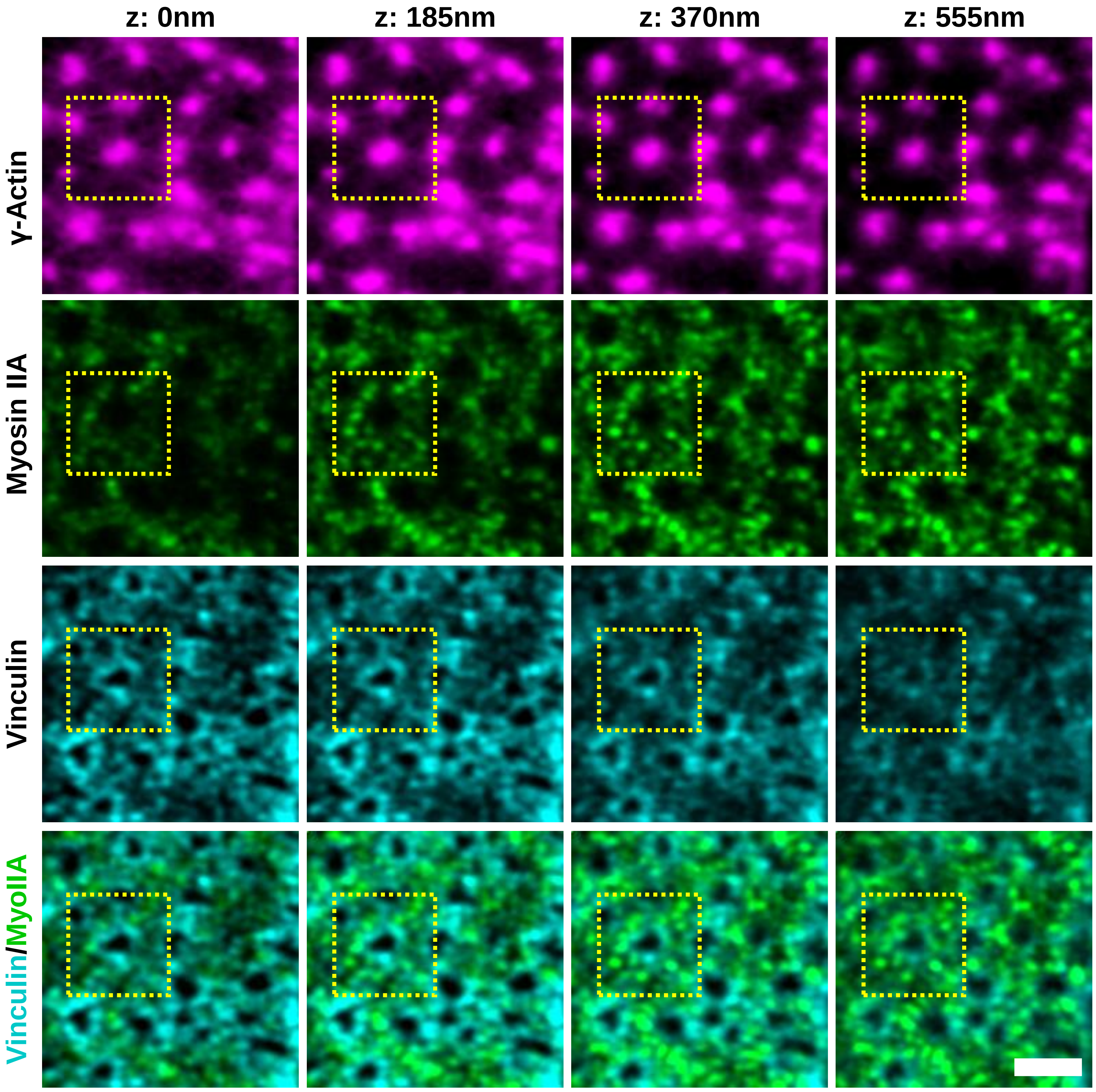

Supplementary Figure 4

Vinculin and myosin IIA occupy distinct regions in podosome clusters
3D-Airyscan images of a DC stained for actin (magenta), myosin IIA (green) and vinculin (cyan). Images shown are the total clusters for the single podosome shown in main Figure 3a. Podosome shown in main Figure 3a is indicated by a the yellow dashed square. Scale bar = 2 µm.

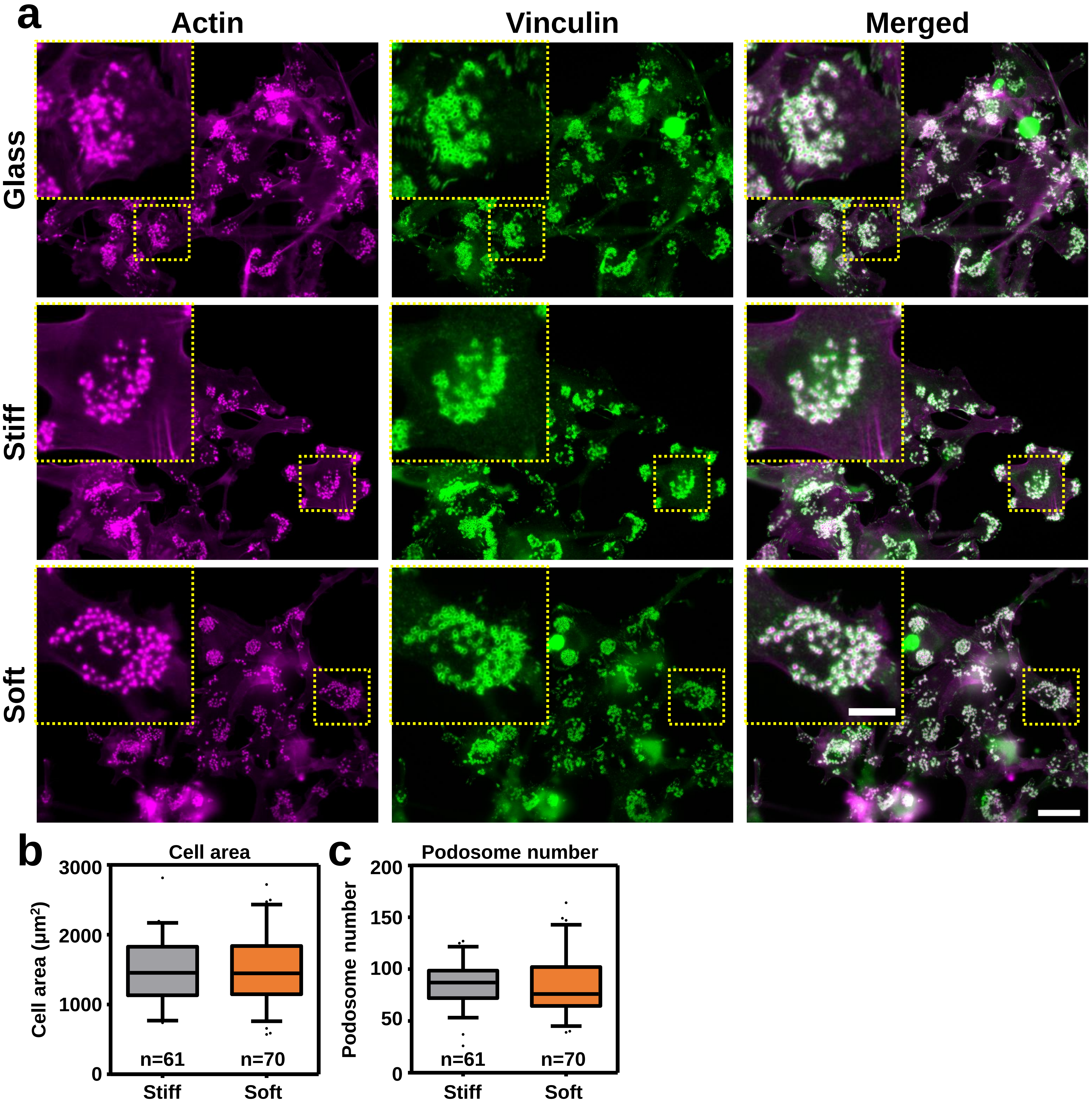

Supplementary Figure 5

DCs spread and form podosomes independent of substrate stiffness

a, DCs were seeded on glass, stiff and soft PDMS and fixed after 3 hrs. Shown are widefield images of DCs stained for actin (magenta) and vinculin (green). Insets depict single cells and the right panel depicts the merged image. **b,** Quantification of cell area on stiff (61 cells) and soft (70 cells) substrates. **c,** Quantification of podosome number on stiff (61 cells) and soft (65 cells) substrates. Scale bar = 25 µm; inset = 10 µm.

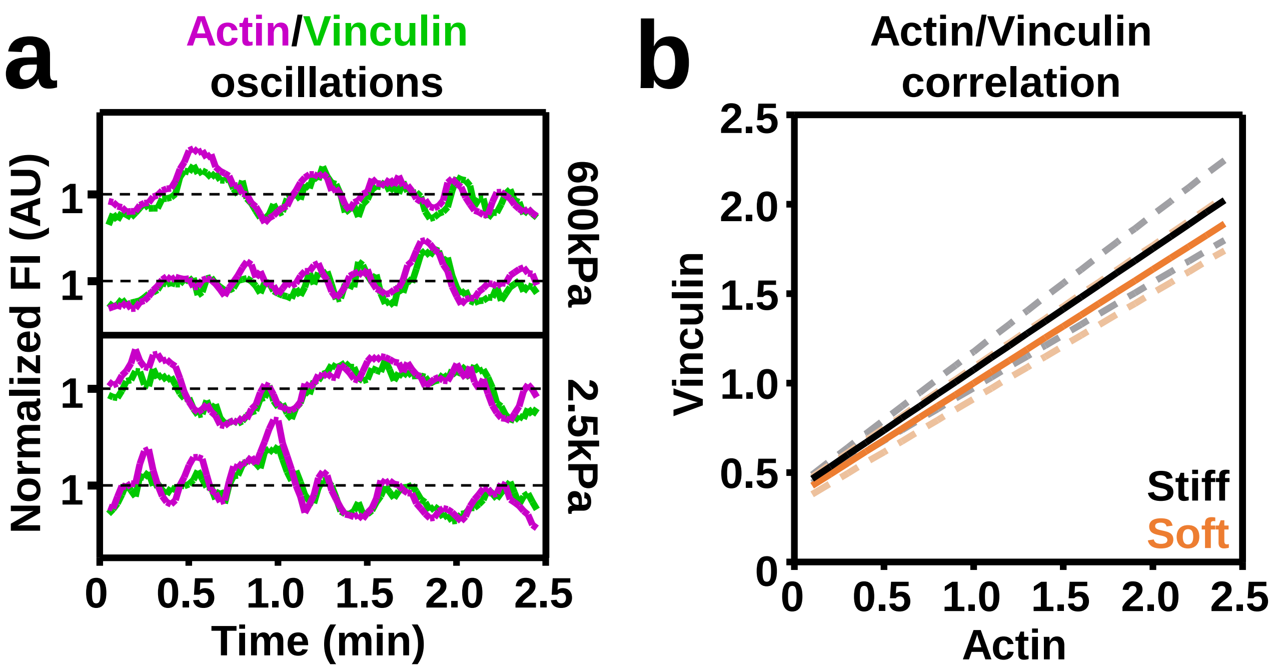

Supplementary Figure 6

Podosome protrusive forces only deform soft PDMS

a, DCs were transfected with Lifeact-GFP and vinculin-mCherry. Imaging was performed with Airyscan confocal microscopy with 15s frame intervals. The graph depicts oscillations of the fluorescence intensity of Lifeact-GFP (magenta) and vinculin-mCherry (green) in representative podosomes on stiff (upper panel) and soft (lower panel) substrate over time. **b,** Lifeact-GFP and vinculin-mCherry fluorescence intensity was correlated and fitted using a linear regression analysis on both stiff (r^2=0.51, P<0.0001) and soft (r^2=0.77, P<0.0001) substrates.

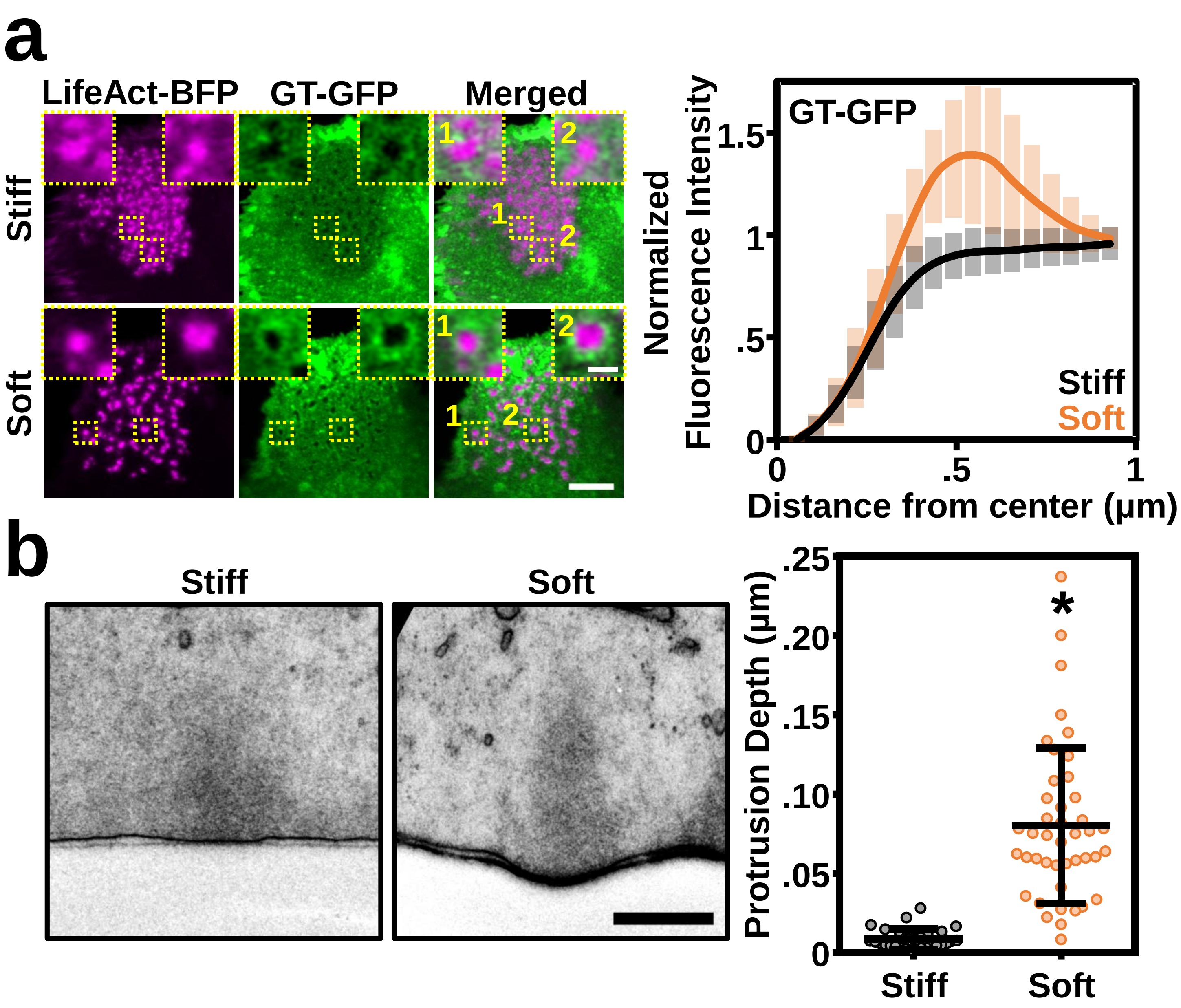

Supplementary Figure 7

Podosome protrusive forces only deform soft PDMS

a, Airyscan images of DCs transfected with LifeAct-BFP and GT-GFP. The graph shows the average radial fluorescent intensity profile of GT-GFP on stiff (black) and soft (orange) substrates. **b,** Transverse EM sections of single podosomes showing no protrusion on the stiff substrate and a small protrusion on the soft substrate. The graph depicts a quantification of the protrusion depth on both stiff and soft substrates. Scale bars: **a** = 5 µm, **b** = 0.5 µm; inset **a** = 1 µm.

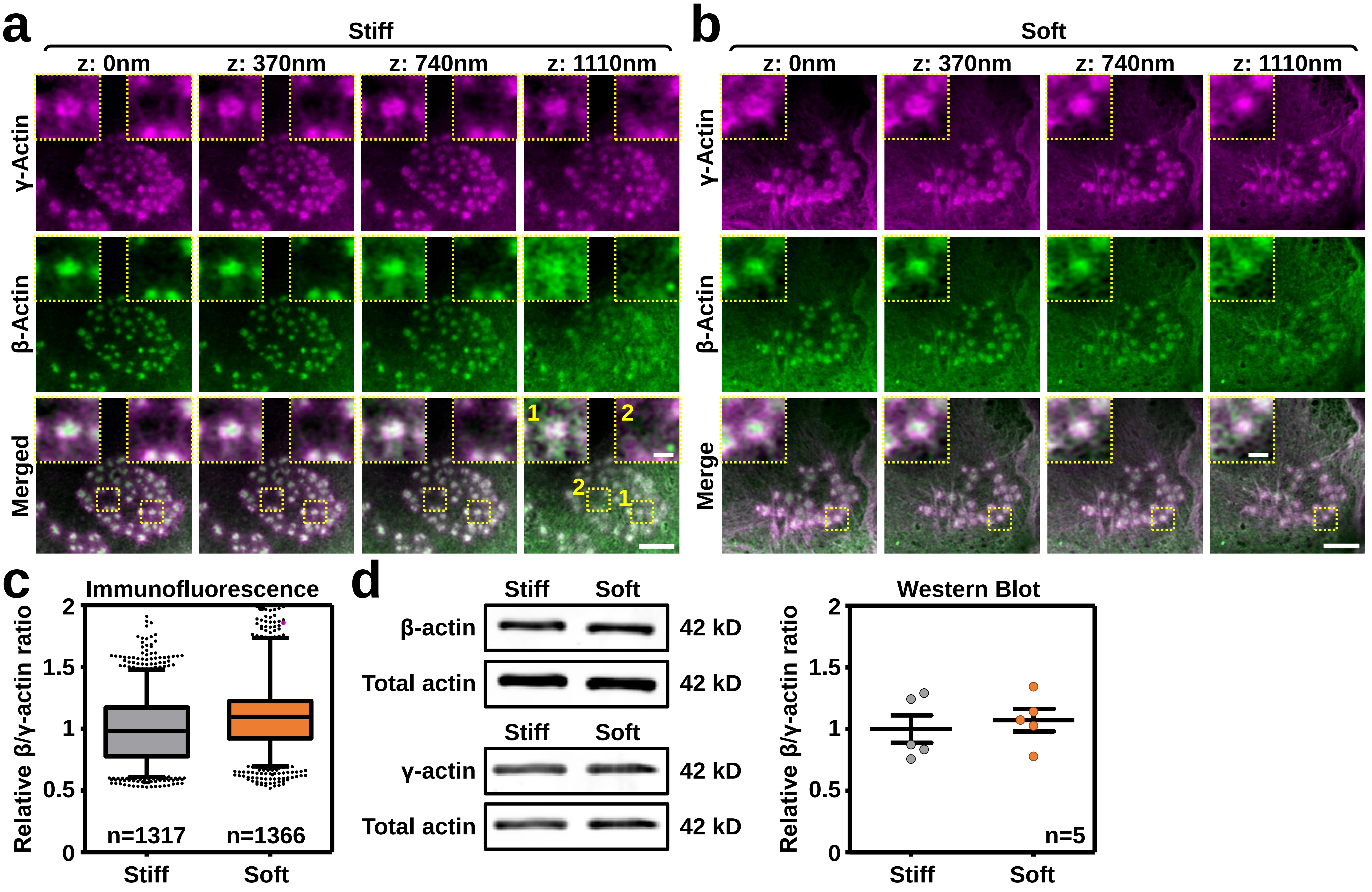

Supplementary Figure 8

Differential localization and relative levels of β and γ-actin are independent of substrate stiffness
a-b, 3D-Airyscan images of DCs seeded on **a**, stiff and **b**, soft substrates and stained for γ (magenta) and β-actin (green). Insets depict a single podosome. Note the localization of γ-actin to both the pPM (inset 1) and the radiating filaments (inset 2) on stiff substrates. **c,** Quantification of the β/γ actin ratio on soft (n = 1317 podosomes) and stiff (n = 1366 podosomes) substrates. **d,** Shown is a western blot analysis of β and γ-actin of lysates prepared from VPMs of DCs on stiff and soft substrates. Total actin is used as a loading control. The full membrane is shown in Supplementary Fig. 18. The graph depicts a quantification of the β/γ actin ratio in 5 independent experiments. Scale bars: **a,b** = 5 µm; insets: **a,b** = 1 µm.

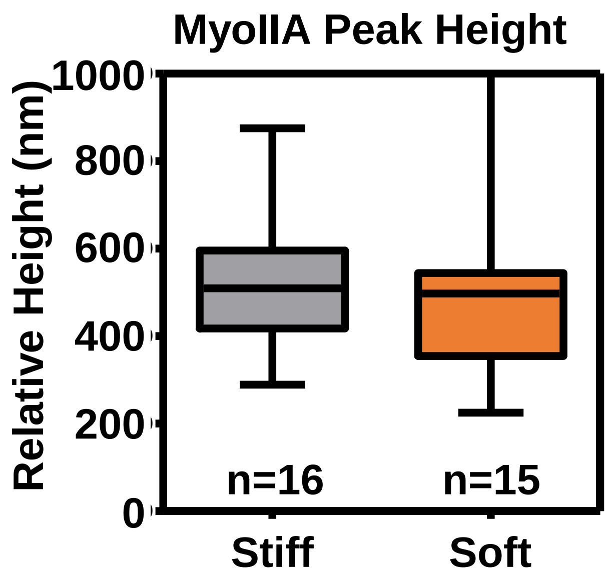

Supplementary Figure 9

Myosin IIA peak intensity not controlled by substrate stiffness.
Quantification of the fluorescence peak height of myosin IIA on stiff (n=16 clusters) and soft (n = 15 clusters) substrates.

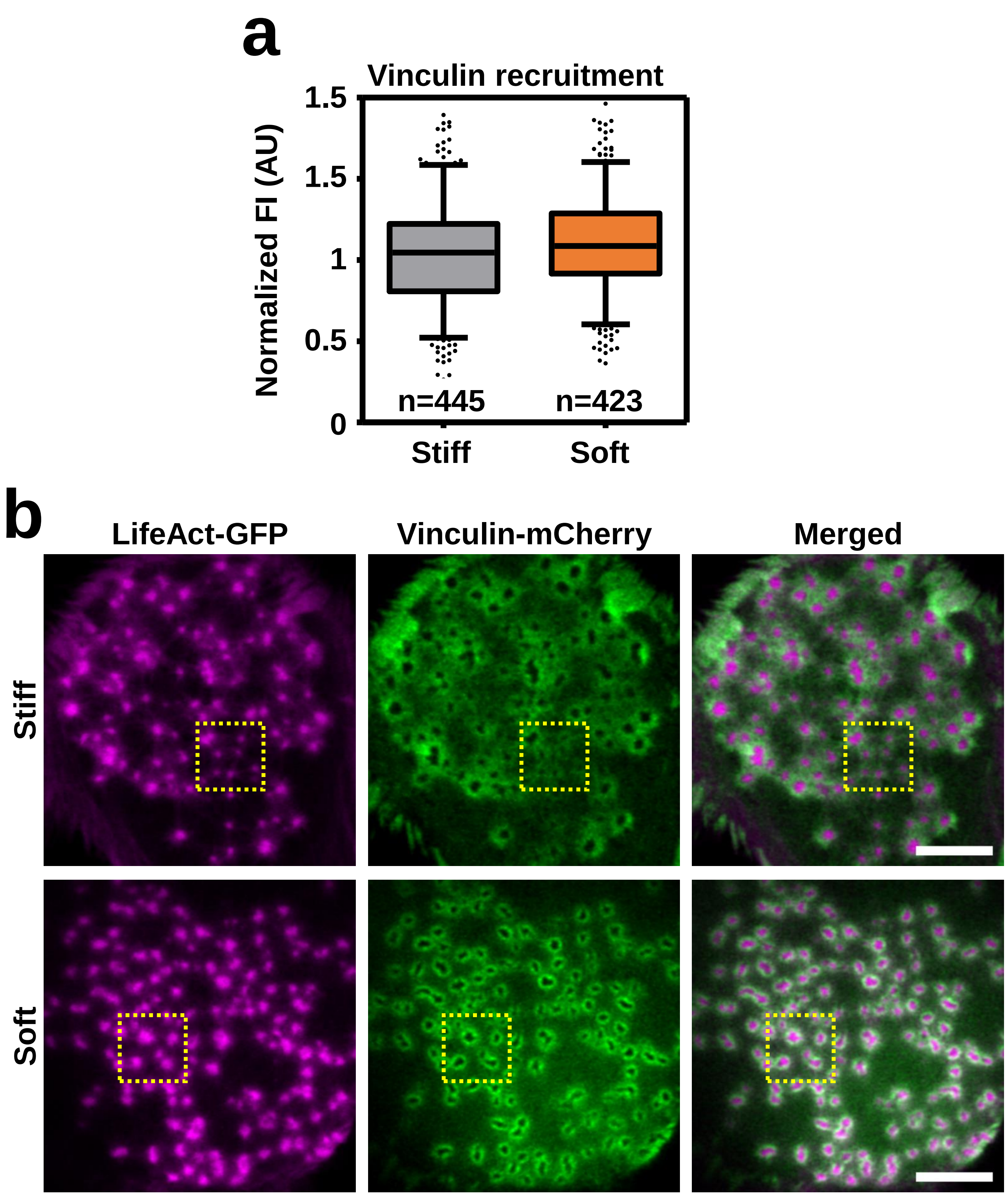

Supplementary Figure 10

Actin and vinculin reorganize on soft substrates

a, Quantification of vinculin recruitment to podosomes on stiff (n=445) and soft substrates (n=423 podosomes) by measuring the fluorescence intensity of vinculin around podosome cores. **b,** Airyscan images of living DCs transfected with Lifeact-GFP (magenta) and Vinculin-mCherry (green) and seeded on stiff and soft substrates. The yellow dashed square indicates the area depicted in main Figure 6c. FI=fluorescence intensity. Scale bar = 5 µm.

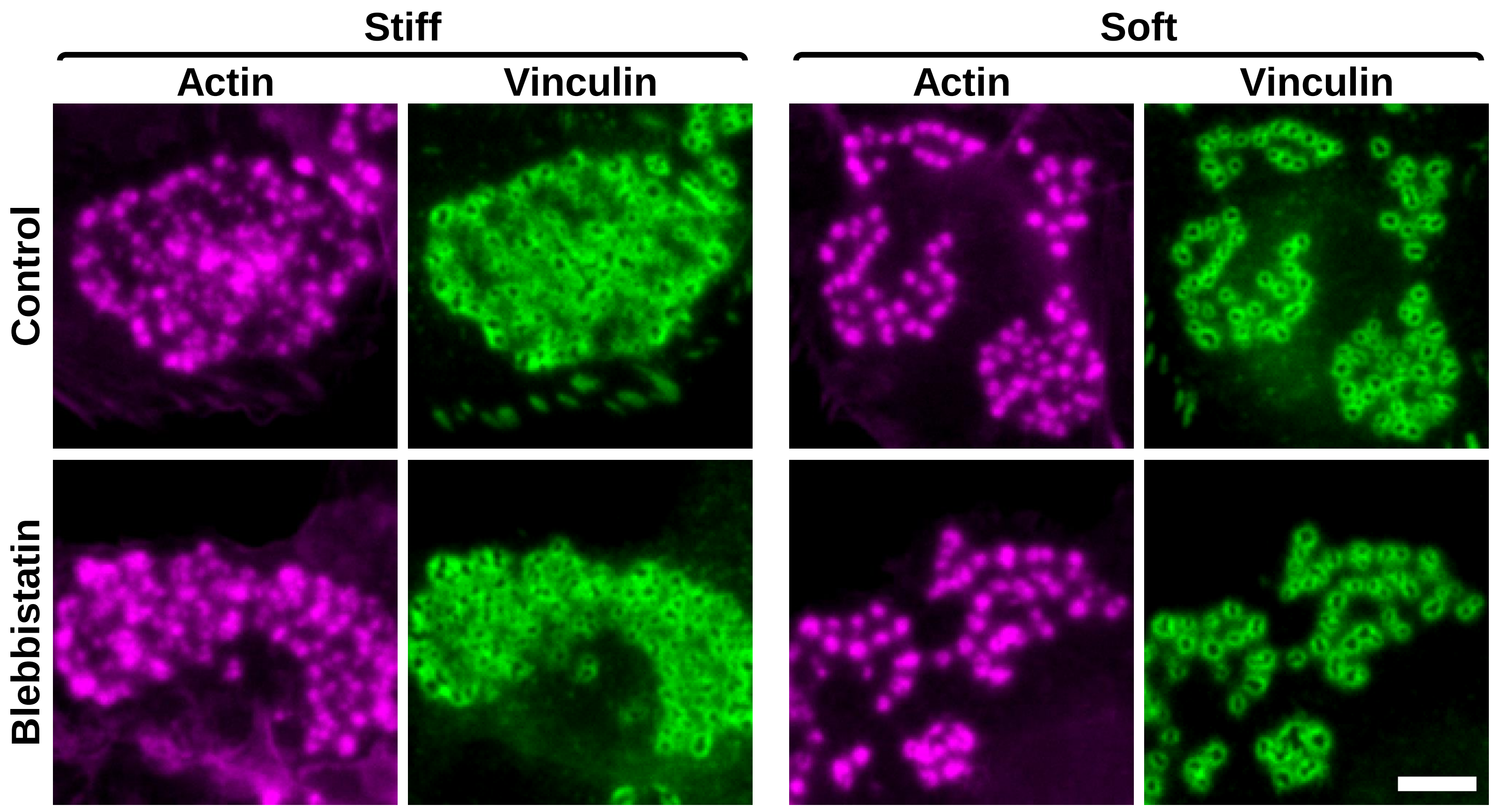

Supplementary Figure 11

Myosin IIA inhibition does not control the stiffness induced relocalization of vinculin
Widefield images of DCs seeded on stiff and soft substrates, fixed and stained for actin (magenta) and vinculin (green). Cells were either left unstimulated (top panels) or treated with 50 μM Blebbistatin for 1 hr (bottom panels) prior to fixation. Scale bar = 5 μm.

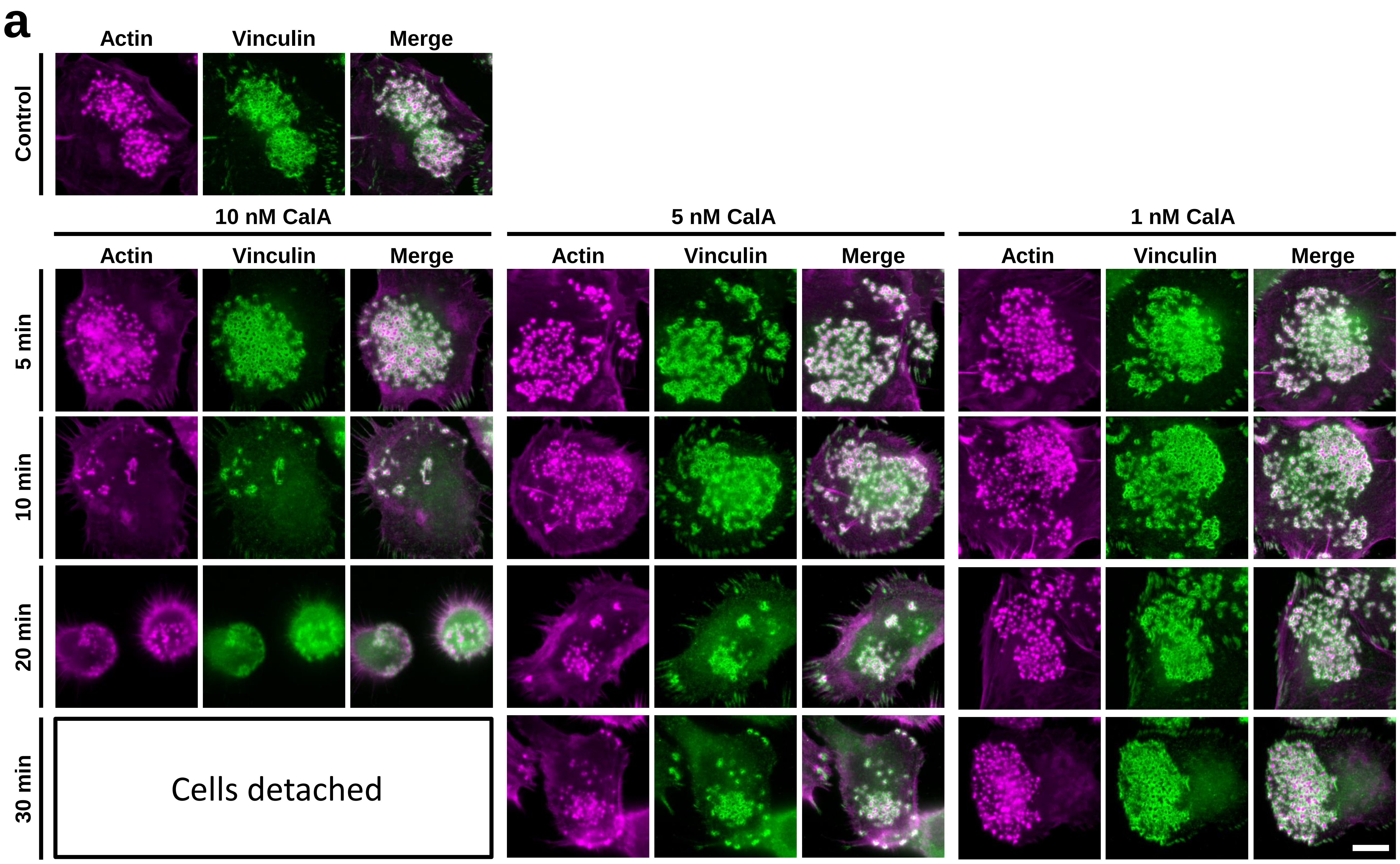

Supplementary Figure 12 continues on the next page

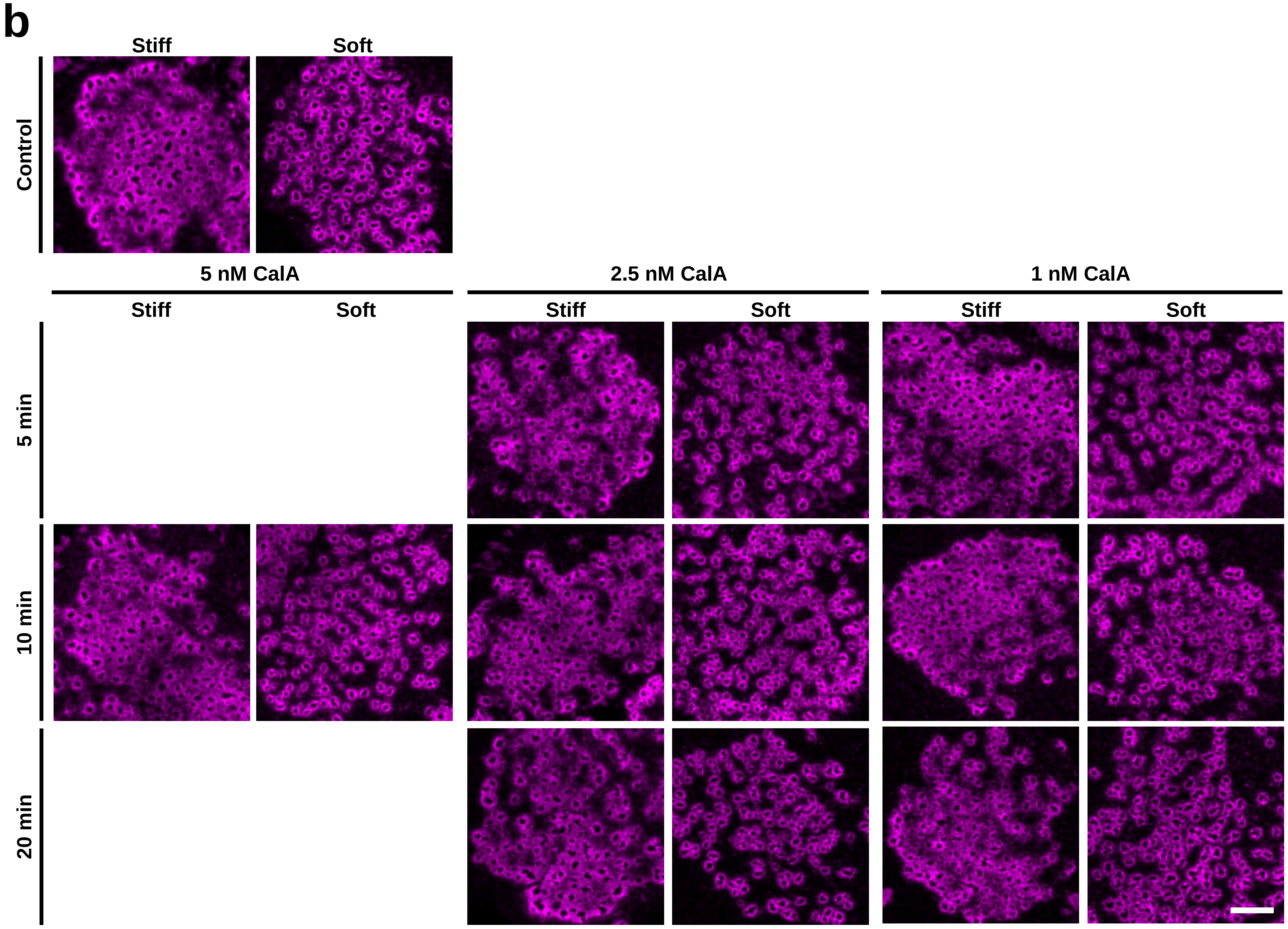

Supplementary Figure 12

Myosin IIA activation does not affect the stiffness induced relocalization of vinculin
a, Widefield images of DCs seeded on glass coverslips, fixed and stained for actin (magenta) and vinculin (green). Cells were either left unstimulated (control) or treated with Calyculin A (CalA) for the indicated times and with the indicated concentrations prior to fixation. **b,** Airyscan images of DCs seeded on stiff and soft substrates, fixed and stained for vinculin (magenta). Cells were either left unstimulated (control) or treated with Calyculin A (CalA) for the indicated times and with the indicated concentrations prior to fixation. Scale bars: **a** = 10 µm, **b** = 5 µm.

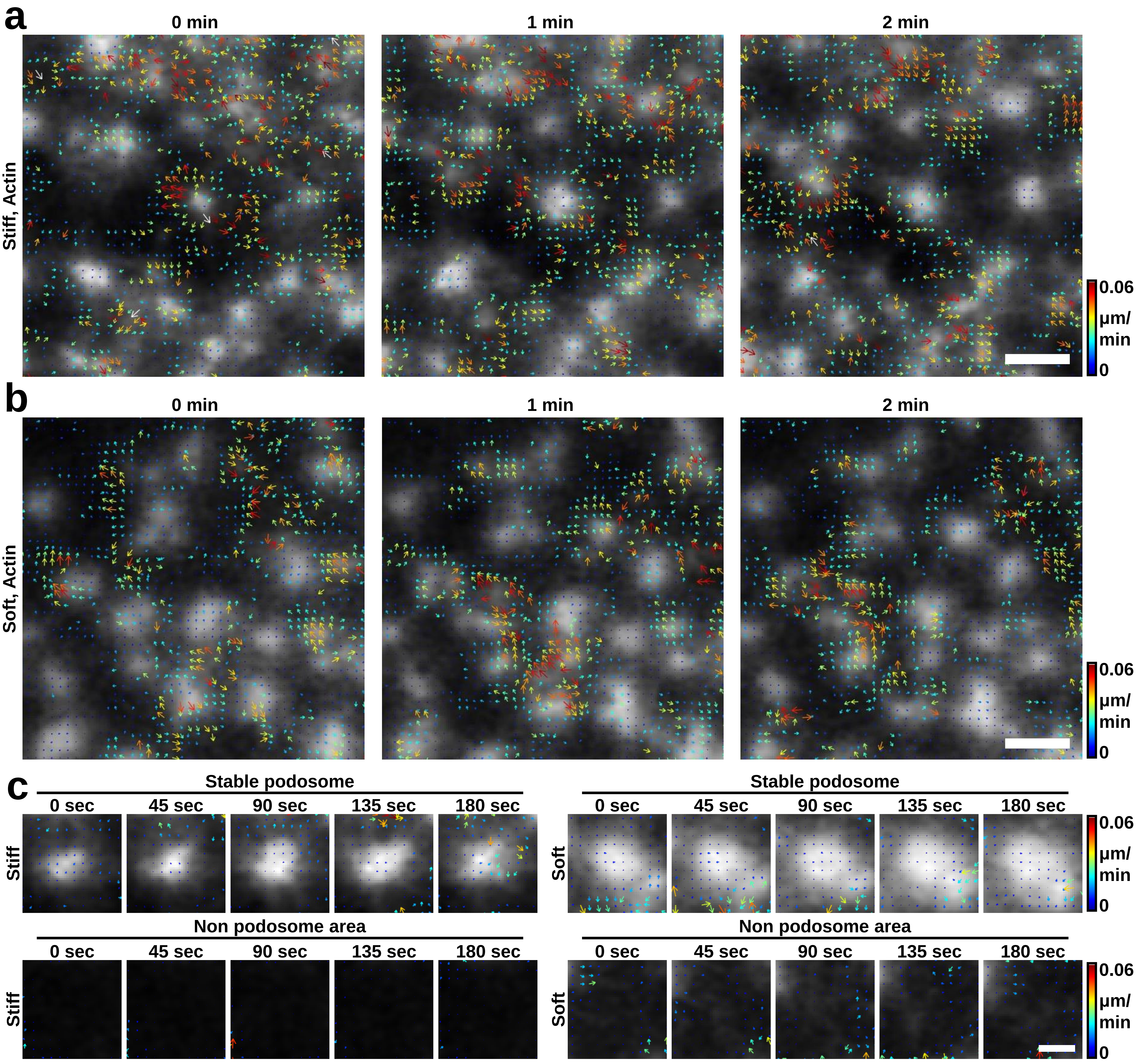

Supplementary Figure 13

twSTICS reveals flows of actin in dynamic podosomes on stiff and soft substrates

a-c, DCs were transfected with Lifeact-GFP and vinculin-mCherry. Airyscan imaging was performed with 15s frame intervals. Time series were subjected to twSTICS analysis and results are plotted as vector maps in which the arrows indicate direction of flow and both the size and color are representative of the flow magnitude. Shown are representative waves of vectors for actin on **a,** stiff and **b,** soft substrates as well as in **c,** stable and non podosome areas. Scale bars: **a,b** = 1 μm, **c** = 0.5 μm.

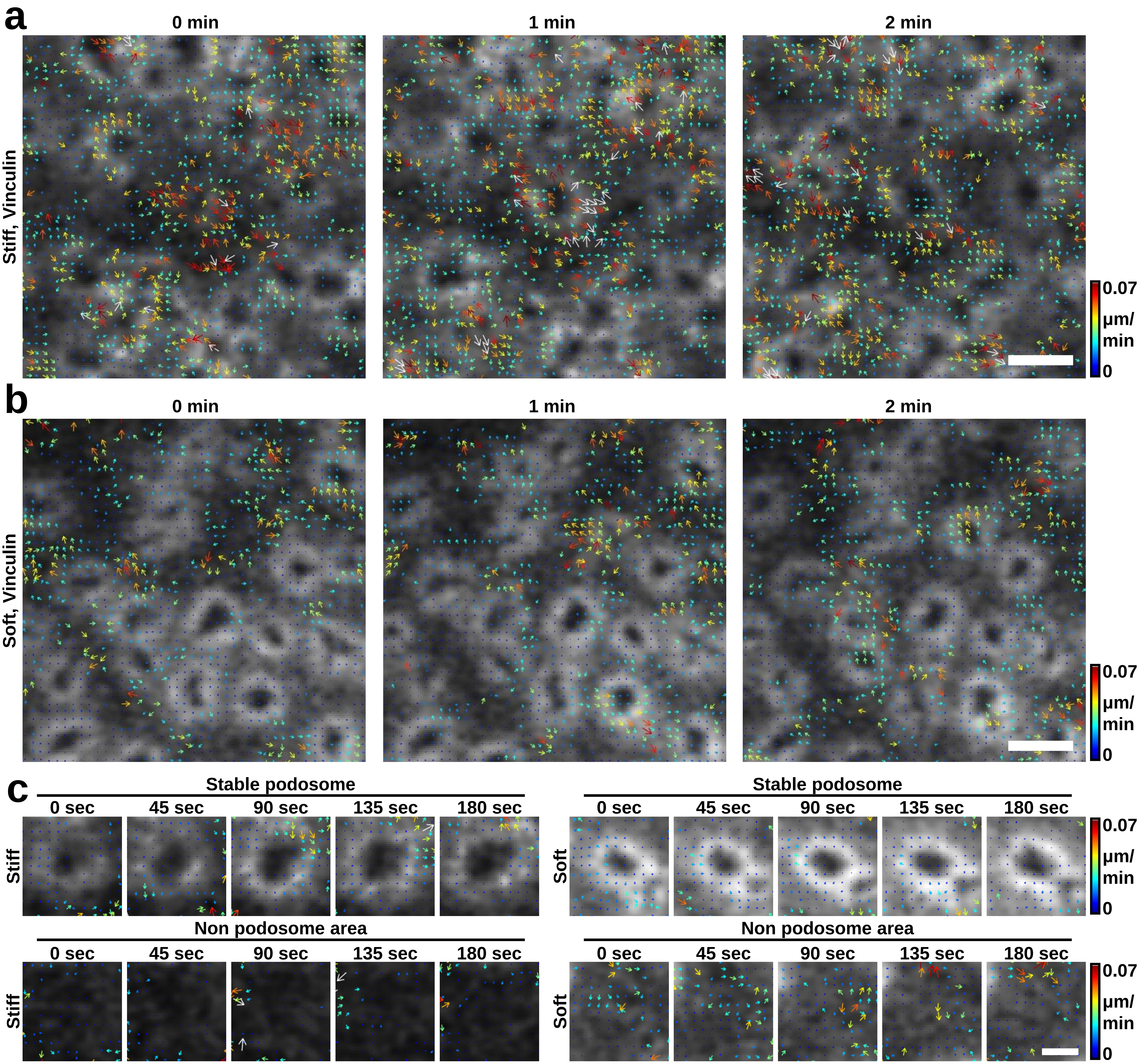

Supplementary Figure 14

twSTICS reveals flows of vinculin in dynamic podosomes on stiff and soft substrates

a-c, DCs were transfected with Lifeact-GFP and vinculin-mCherry. Airyscan imaging was performed with 15s frame intervals. Time series were subjected to twSTICS analysis and results are plotted as vector maps in which the arrows indicate direction of flow and both the size and color are representative of the flow magnitude. Shown are representative waves of vectors for vinculin on **a,** stiff and **b,** soft substrates as well as in **c,** stable and non podosome areas. Scale bars: **a,b** = 1 µm, **c** = 0.5 µm.

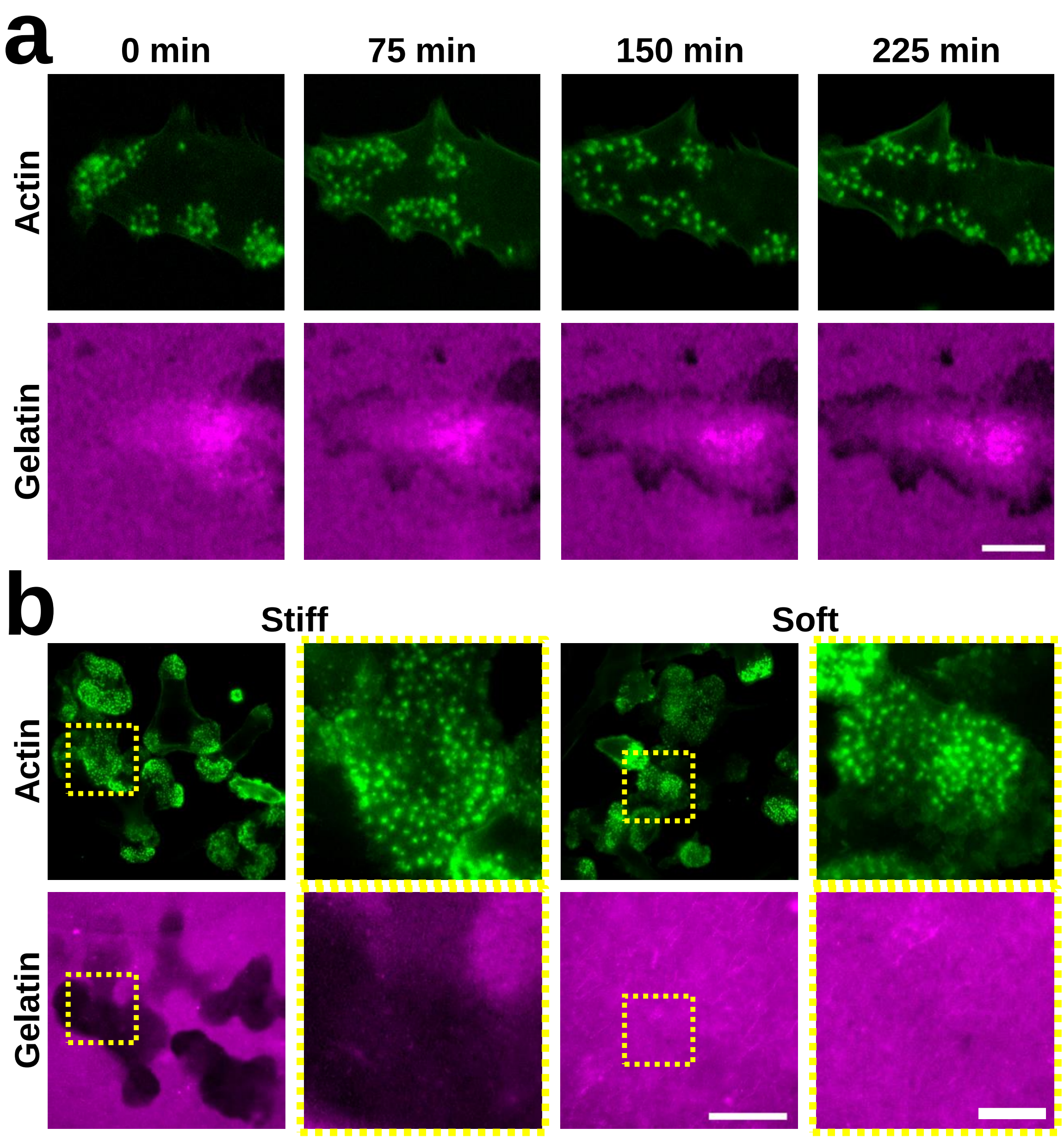

Supplementary Figure 15

Decreased gelatin degradation on soft substrates

a, DCs were transfected with Lifeact-GFP (green) and seeded on stiff PDMS that as coated with rhodamine-conjugated gelatin (magenta). Image series were acquired overnight on a Leica DM6000 widefield microscope with 1 image per 5 min. Shown are stills from the image series demonstrating the specific degradation of gelatin underneath areas with podosome formation (See also Supplementary Video 6). **b**, DCs were seeded on gelatin-rhodamine (magenta) coated stiff and soft substrates, incubated overnight and subsequently stained for actin (green). Shown are representative images of gelatin degradation of stiff (right panels) and soft (left panels) substrates. Images in column 1 and 3 are the same as in Fig. 5j. Images in column 2 and 4 depict representative single cells. Scale bars: **a** = 10 µm, **b** = 20 µm; inset: **b** = 5 µm.

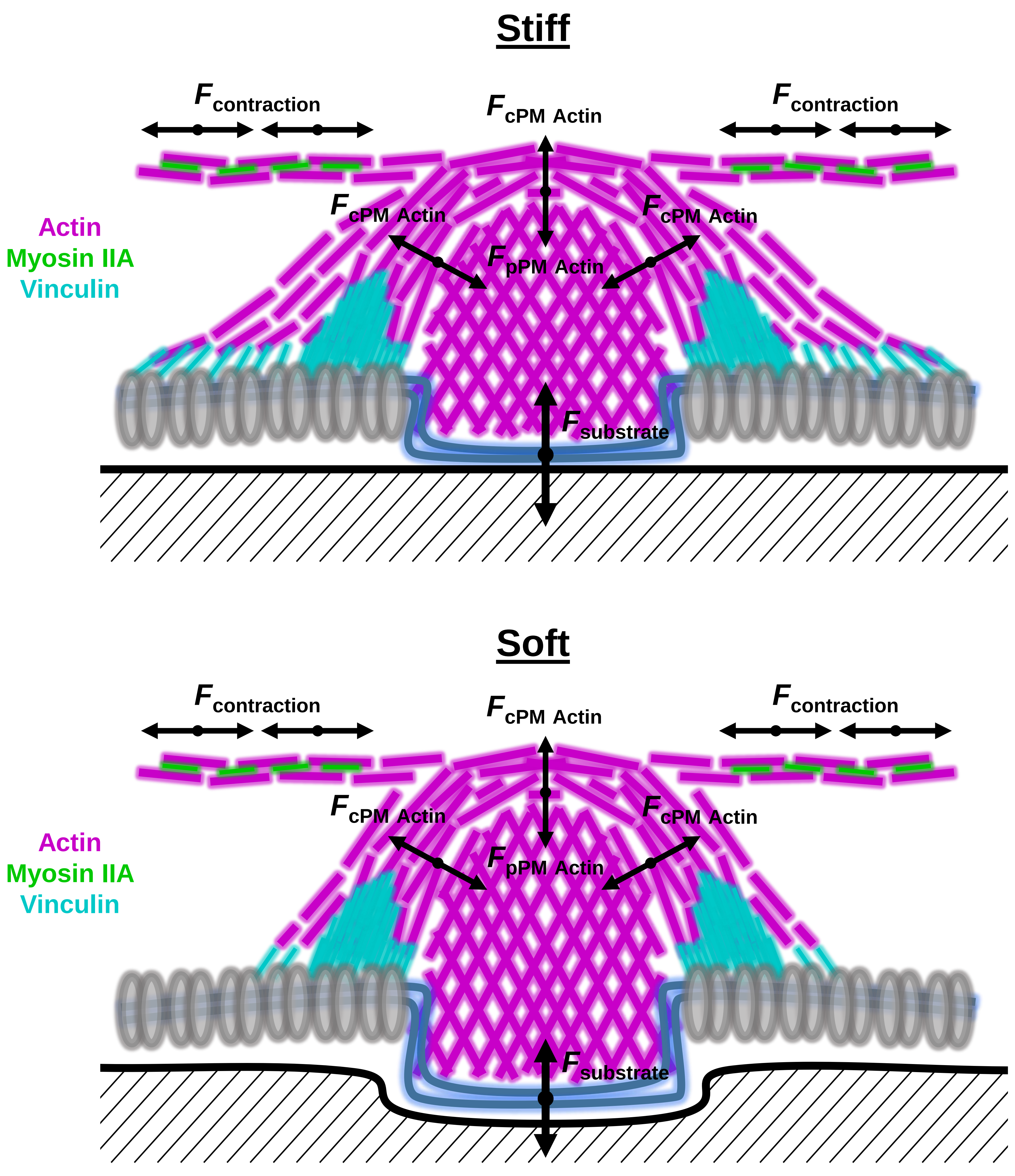

Supplementary Figure 16

Schematic model of how forces may be balanced in the podosome protrusive core
cPM actin polymerization generates a downward protrusive force that is initially counterbalanced by the an upward force from the underlying substrate. The subsequent vertical growth of the cPM actin generates an upward force at the top of podosomes that is balanced by the pPM actin. Myosin IIA in the dorsal actin filaments induces contractile forces, probably to facilitate long range inter-podosomal force connections.

a

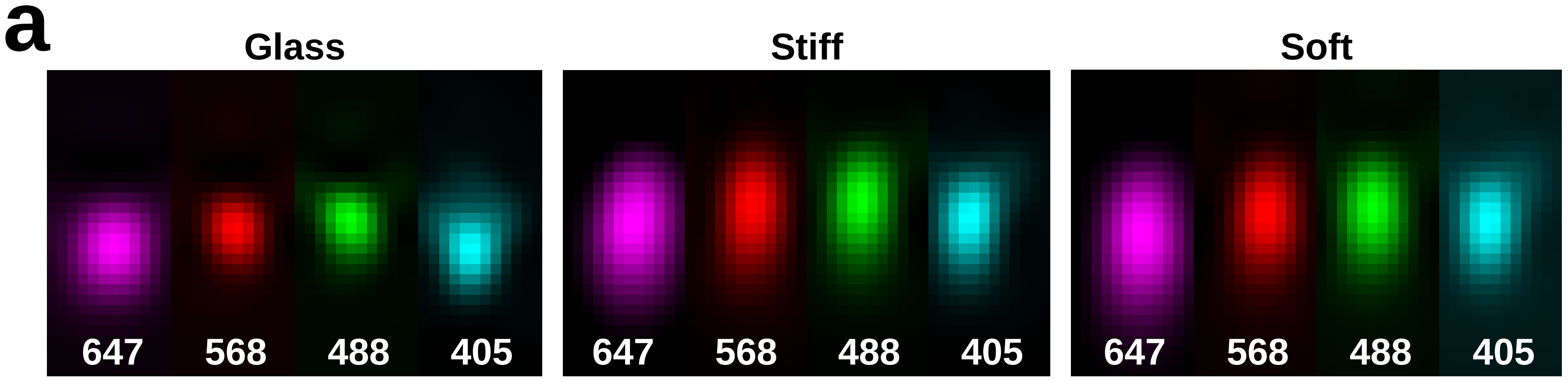

b

Glass

	FWHM (XY)	FWHM (Z)
**405**	185 nm	611 nm
**488**	174 nm	511 nm
**568**	194 nm	522 nm
**647**	265 nm	705 nm

Stiff

	FWHM (XY)	FWHM (Z)
**405**	190 nm	740 nm
**488**	197 nm	818 nm
**568**	199 nm	910 nm
**647**	261 nm	1070 nm

Soft

	FWHM (XY)	FWHM (Z)
**405**	188 nm	741 nm
**488**	194 nm	818 nm
**568**	198 nm	861 nm
**647**	250 nm	1080 nm

Supplementary Figure 17

PSF measurements on glass, stiff and soft PDMS

a, TetraSpeck microspheres of 200 nm labeled with Alexa405, Alexa488, Alexa568 and Alexa647 were left to settle down on glass, stiff and soft PDMS and PSF measurements were collected using Airyscan settings on the LSM 880. Shown are the x-y,z profiles of the beads for each of the four channels. **b,** Quantification of the FWHM values in x-y as well as in z for the four channels on glass, stiff and soft PDMS. Note that the FWHM is not altered by PDMS in x-y but is increased by approximately 1.5 fold in z compared to glass.

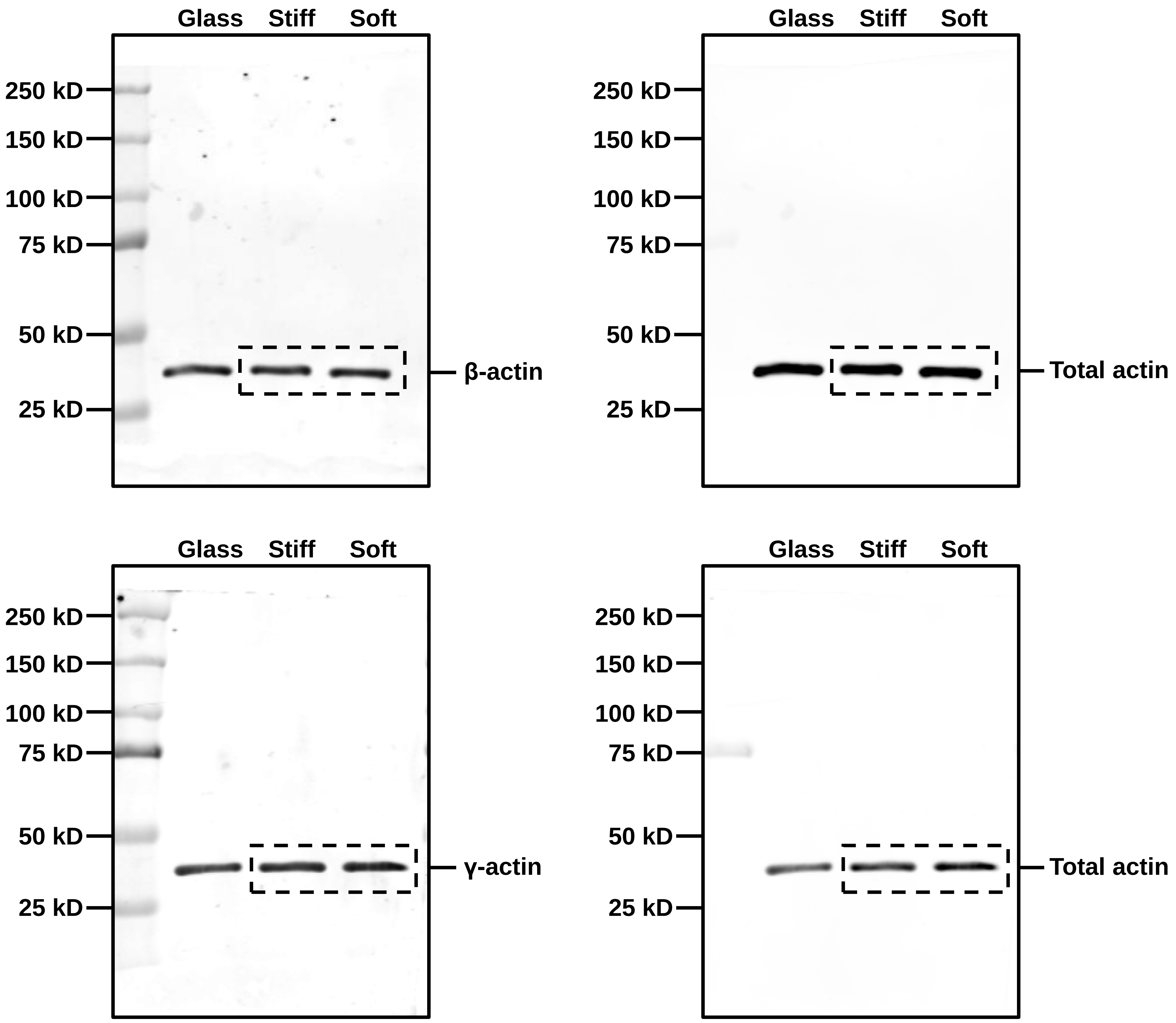

Supplementary Figure 18

Full western blot membranes from supplementary Fig. 8d. Dashed line indicates cropped region shown in Figure.
